# Study protocol for a multicenter randomised controlled trial on the (cost)effectiveness of biopsy combined with same-session MR-guided LITT versus biopsy alone in patients with primary irresectable glioblastoma (EMITT trial)

**DOI:** 10.1186/s12885-023-11282-7

**Published:** 2023-08-23

**Authors:** Céline L.G. Neutel, Ilaria Viozzi, Christiaan G. Overduin, Anne Rijpma, Janneke P.C. Grutters, Gerjon Hannink, Pieter van Eijsden, Pierre A. Robe, Maroeska M. Rovers, Mark ter Laan

**Affiliations:** 1 Department of Neurosurgery, Radboud University Medical Center, Nijmegen, The Netherlands; 2 Department of Medical Imaging, Radboud University Medical Center, Nijmegen, The Netherlands; 3 Department for Health Evidence, Radboud University Medical Center, Nijmegen, The Netherlands; 4 Department of Neurosurgery, University Medical Center Utrecht, Utrecht, The Netherlands

**Keywords:** Glioblastoma, Laser ablation, Laser therapy, Ablation, Brain neoplasms, Magnetic resonance-guided laser-induced thermal therapy

## Abstract

**Background:**

Glioblastoma (GBM) is the most common primary, malignant brain tumour with a 5-year survival of 5%. If possible, a glioblastoma is resected and further treated with chemoradiation therapy (CRT), but resection is not feasible in about 30% of cases. Current standard of care in these cases is a biopsy followed by CRT. Magnetic resonance (MR) imaging-guided laser interstitial thermal therapy (LITT) has been suggested as a minimally invasive alternative when surgery is not feasible. However, high-quality evidence directly comparing LITT with standard of care is lacking, precluding any conclusions on (cost-)effectiveness. We therefore propose a multicenter randomized controlled study to assess the (cost-)effectiveness of MR-guided LITT as compared to current standard of care (EMITT trial).

**Methods and analysis:**

The EMITT trial will be a multicenter pragmatic randomized controlled trial in the Netherlands. Seven Dutch hospitals will participate in this study. In total 238 patients will be randomized with 1:1 allocation to receive either biopsy combined with same-session MR-guided LITT therapy followed by CRT or the current standard of care being biopsy followed by CRT. The primary outcomes will be health-related quality of life (HR-QoL) (non-inferiority) using EORTC QLQ-C30 + BN20 scores at 5 months after randomization and overall survival (superiority). Secondary outcomes comprise cost-effectiveness (healthcare and societal perspective) and HR-QoL of life over an 18-month time horizon, progression free survival, tumour response, disease specific survival, longitudinal effects, effects on adjuvant treatment, ablation percentage and complication rates.

**Discussion:**

The EMITT trial will be the first RCT on the effectiveness of LITT in patients with glioblastoma as compared with current standard of care. Together with the Dutch Brain Tumour Patient association, we hypothesize that LITT may improve overall survival without substantially affecting patients’ quality of life.

**Trial registration:**

This trial is registered at ClinicalTrials.gov (NCT05318612).

**Supplementary Information:**

The online version contains supplementary material available at 10.1186/s12885-023-11282-7.

## Introduction

Glioblastoma (GBM) is the most common and most malignant primary brain tumour [1]. These tumours account for around 60% of all primary brain tumours in adult patients with an incidence ranging from 3.9 to 4.17 per 100.000 persons [2]. GBMs are in most cases highly debilitating and often occur at a relatively young age (median 59 years) [3]. Although many efforts have been made to improve current therapies, patients with GBM face a poor prognosis, with a 5-year survival of 5% [4, 5]. Current standard of care includes surgical resection followed by adjuvant chemoradiation therapy (CRT) [6]. This strategy has been associated with median overall survival (OS) of 14.5–18.5 months, depending on the extent of resection [7, 8]. However, in 30% of patients surgery appears not to be feasible [9], most often due to the unfavourable location of the tumour, and sometimes because the patient does not desire surgical resection due to the possible complications and/or risk of further deterioration. These patients miss the benefit of surgical resection since biopsy followed by CRT results in a faster decline in quality of life and a median survival ranging from 5 to 9.4 months [10]. Laser-induced thermal therapy (LITT) (also referred to as Stereotactic Laser Ablation (SLA)), has been developed as a minimally invasive treatment for tumours, including brain tumours [11]. A recent systematic review, consisting of phase I and II studies, showed that the median survival in patients with newly diagnosed irresectable glioblastoma who received LITT is 10.2 months [12]. No data regarding the quality of life of patients who have undergone LITT is available. To date, no randomized controlled clinical trials looking at the effectiveness of LITT in patients with newly diagnosed irresectable glioblastoma have been published. A search on clinicaltrials.gov, ISRCTN Registry and EU clinical trials register for (ongoing) trials identified no (planned) randomized studies investigating the role of LITT in patients with newly diagnosed irresectable glioblastoma, precluding any conclusions on (cost-)effectiveness.

A preceding pilot study at Radboudumc [13] including 15 patients of which 10 treated with LITT showed that patients with an irresectable glioblastoma are willing to participate in a randomized trial. In addition, safety and feasibility data are acceptable and in line with previous reports [11, 14].

Given the potential survival benefit but lack of evidence to support LITT, we will conduct a multicenter randomized controlled trial to evaluate effectiveness of this technique in patients with primary irresectable glioblastoma (EMITT trial). It is expected that addition of LITT will yield longer survival without substantially affecting patients’ quality of life. Only when the survival is superior and the quality of life is non-inferior in the intervention group compared to the control group, LITT will be considered effective. Furthermore, due to the increasing pressure on healthcare budgets, cost-effectiveness of LITT will also be assessed.

## Methods

### Study design

The EMITT trial is designed as a multicenter pragmatic randomized controlled trial. Patients will be randomized with 1:1 allocation to receive either biopsy followed by adjuvant chemotherapy and/or radiation which is the current standard of care (control group), or biopsy combined with same-session MR-guided LITT therapy followed by adjuvant chemotherapy and/or radiation (intervention group). The trial is registered at www.clinicialtrials.gov (NCT05318612).

### Study setting

Seven Dutch hospitals will participate in this trial (Appendix 1). Six of the seven hospitals are academic hospitals, one hospital is a non-academic hospital. Biopsy combined with same-session LITT therapy will be performed in two designated and trained centers, namely the Radboud university medical center (Nijmegen) and the University Medical Center Utrecht (Utrecht). Biopsy alone will be performed at all including participating centers. Adjuvant treatment will be given either in the including center or in a local hospital in the patient’s own region.

### Study population

Patients with a suspected diagnosis of glioblastoma will be discussed at their local multidisciplinary tumor board meeting. When the board advises biopsy only due to the unfavorable location of the tumor, or if the patient wishes no resection, the subject is potentially eligible for this study. The local participating neurosurgeon will then discuss the case with the centralized study expert panel. This will be done by sharing anonymized, necessary clinical and radiological information through a safe digital environment. The expert panel consists of three neurosurgeons, involved in the trial. The expert panel will review all information to judge if the patient is a potential candidate for LITT according to the inclusion criteria. An overview of in- and exclusion criteria is provided in Table 1. When eligible, the patient will be informed about the study by one of the members of the study team both orally and in writing through the patient information form (PIF). After a mandatory reflection period of 48 h, the patient will be called by one of the researchers and will be asked whether he or she wishes to participate in the study. After having obtained written informed consent, the patient will be formally included in the study and will be randomized.

**Table 1:** Overview of in- and exclusion criteria

Inclusion criteria	Exclusion criteria
• Suspected glioblastoma	• Pregnancy
• Supratentorial localization of the tumor	• Contraindication for general anesthesia or MRI
• Age over 18 years	• Final diagnosis other than glioblastoma
• Patient is not amenable for surgical resection • Karnofsky Performance Status > = 70	• Insufficient command of the Dutch language by the patient or a family member, making it impossible to fill in the questionnaires

#### Glioblastoma

Whenever the term “glioblastoma” or “GBM” is used in this protocol, it refers to all WHO grade 4 gliomas of the new 2021 classification [12]. Therefore, this includes:

- Glioblastoma, IDH wildtype, WHO grade 4;
- Diffuse hemispheric glioma, H3,3 G34-mutant, WHO grade 4;
- Diffuse midline glioma, H3 K27M-mutant, WHO grade 4;
- Astrocytoma, IDH-mutant, WHO grade 4.

### Randomization and study groups

Randomization will be performed by one of the study physicians using randomized permuted blocks of 4 and 6 through a web-based module (CastorEDC) which is available 7 days a week, 24 h per day. The randomization sequence is concealed and cannot be altered or predicted during the study. Assignments will be balanced in a 1:1 ratio between the two study arms, stratified by center. In the intervention arm, a maximum number of patients treated by each of the LITT centers will be set to 63 to avoid an uneven distribution of patients over the two LITT centers and to share the patient load.

Randomization will be done pre-operatively. It is not deemed feasible to randomize peri-operatively, since LITT requires specific hospital resources (staff, specific operation room and MRI time) which will need to be planned in advance and preferably within two weeks. The study will be open due to practicality and logistic reasons since patients from the intervention group need to be referred to one of the two LITT centers.

After inclusion, the patient will undergo biopsy or biopsy combined with same-session LITT within a maximum of 3 weeks.

### Intervention group

Patients will be admitted the day before the procedure and neuronavigation imaging will be performed. An ablation plan will be made based on the navigation MRI, where one or more trajectories are designed to optimize coverage of the lesion shape while avoiding intervening structures, where planned coverage should be at least 70%.

Patients will receive 4 mg dexamethasone the day before surgery and 8 mg from the day of surgery onward to reduce post-operative edema. Dexamethasone will be phased out after surgery.

LITT procedures will be performed by for LITT trained neurosurgeons. The neurosurgeons who perform LITT in this trial should have performed at least 5 proctored LITT procedures before they are allowed to carry out the procedure independently. The procedure will be performed under general anesthesia. Through a short skin incision, a small twist-drill burr hole and durotomy are performed. Stereotactic biopsy will be performed conform routine procedure using Brainlab neuronavigation and Varioguide™ arm (BrainLAB AG, Feldkirchen, Germany). In the LITT group, frozen section analysis will be performed to confirm diagnosis. If pathological analysis may suggest another diagnosis than a glioma, LITT will not be performed, and the procedure will be terminated.

Upon confirmation of glioma diagnosis, the laser fibers will be inserted and secured with a skull anchor screw. The patient will be transported to the MRI suite where the cooling lines and laser fiberoptic are connected through a waveguide to the control room. Pre-treatment T1-weighted images are acquired to verify the position(s) of the probe(s). For treatment monitoring, MR thermometry images are continuously acquired during laser delivery. LITT will be delivered using a CE-marked MR-guided LITT system for neurosurgical application. Currently, the Visualase Thermal Therapy System (Medtronic, USA), used in this trial, is the only available certified system in Europe for neurosurgical ablation. A Medtronic representative will be present during LITT procedures for technical support. At the end of treatment, contrast-enhanced T1-weighted imaging after intravenous gadolinium injection are acquired. The bone anchor, applicator, and head frame will be removed, and the wound will be sutured.

In case of significant bleeding after biopsy (volume of the hematoma larger than the volume of the tumor on the navigation MRI or diameter of the hematoma larger than 30 mm), the ablation will not be carried out in the trajectory where the bleeding occurred and, if applicable, will only be carried out in the remaining trajectories if the expected total ablation rate is still greater than or equal to 70%.

### Control group

Patients will be admitted the day before surgery and neuronavigation imaging will be made. All neurosurgeons in all participating centers will be allowed to perform this procedure in the context of the trial. Biopsy will be performed under general anesthesia conform local routine procedure. After biopsy, the procedure will be ended. Patients in the control group will not receive additional dexamethasone as part of the trial.

#### Adjuvant treatment

Adjuvant radiotherapy and/or chemotherapy will be prescribed according to the EANO guidelines in both study groups [6].

### Outcomes

#### Primary endpoints

Primary outcomes will be overall survival (superiority), and health-related quality of life (non-inferiority) measured at 5 months after randomization using the scores of the EORTC QLQ-C30 + BN20 questionnaire [15, 16].

#### Secondary endpoints

Secondary outcomes will be healthcare costs using iMCQ and iPCQ questionnaires, (generic) health-related quality of life using the Eq. 5D-5 L and the EORTC QLQ C30 + BN20 questionnaires filled in at different time points during the entire duration of the study, progression free survival (defined as no radiological tumour growth) and tumour response by evaluating follow-up MRI scans, disease specific survival, longitudinal effects, effects on adjuvant treatment, ablation percentage and complication rates.

### Follow-up

In both arms, follow-up will comprise standard of care, i.e., follow-up visit every 3 months with an MRI at 3 and 6 months and later if clinically relevant. Patients will be followed until one of the following events occurs: (i) death; (ii) end of study (max 64 months after start of inclusion) is reached; or (iii) patient withdraws informed consent. Quality of life questionnaires (EORTC QLQ-C30 + BN20 and EuroQol 5D-5 L) will be completed at baseline, < 72 h after surgical procedure, monthly for the first six months from randomization, subsequently at 12 and 18 months after randomization and thereafter yearly up to a maximum of 64 months [15–17]. Resource use and productivity losses will be measured monthly for the first six months from randomization, subsequently at 12 and 18 months after randomization and thereafter yearly up to a maximum of 64 months, using the iMTA Medical Consumption Questionnaire (iMCQ) and iMTA Productivity Cost Questionnaire (iPCQ) [18, 19]. Questionnaires will be filled in online through a secure web-based module, if necessary, with the help of a relative or caregiver. Adverse events will be monitored using the questionnaires and electronic patient files.

**Fig. 1:**
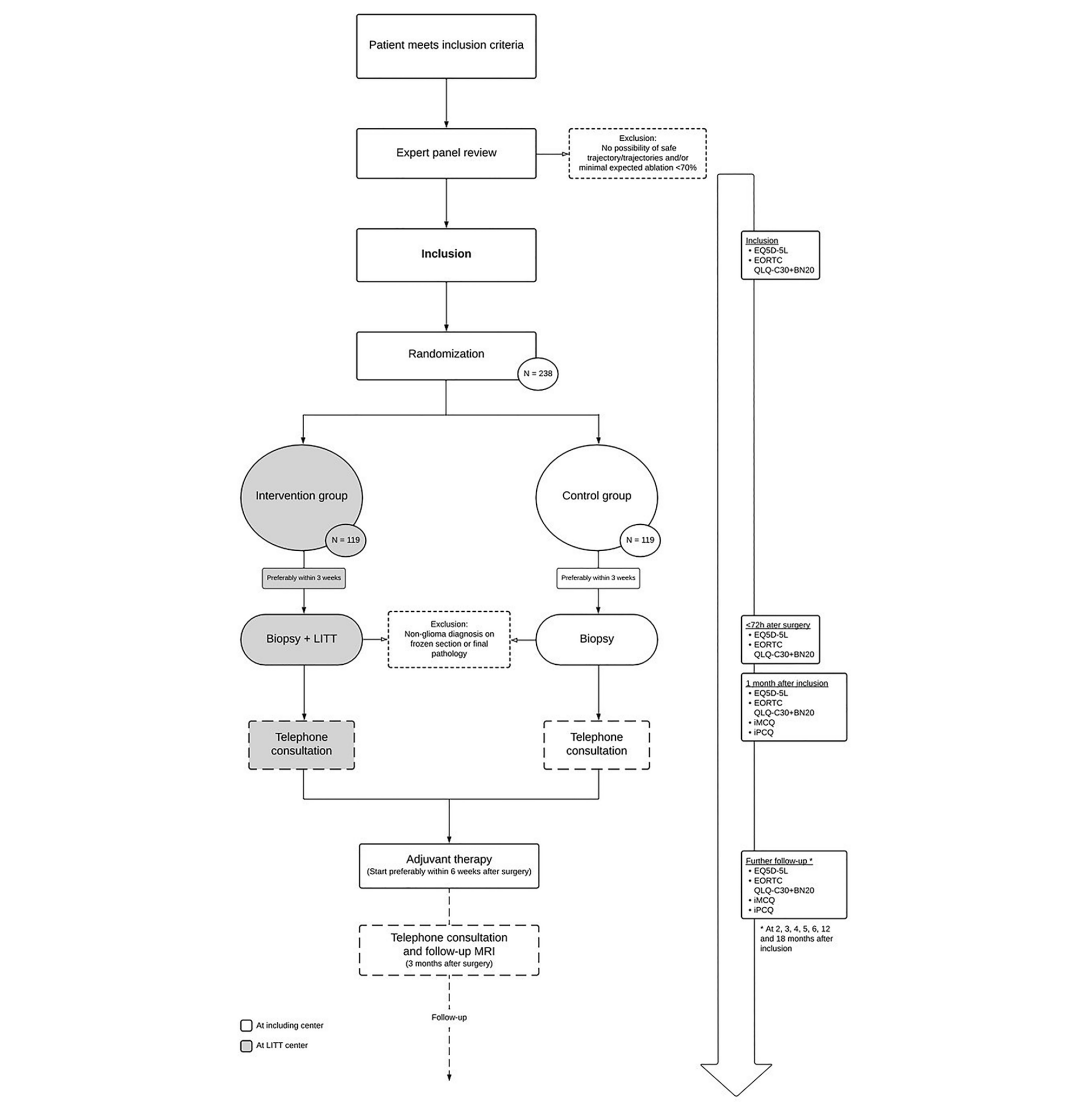
Flowchart of study design

A detailed study flowchart is outlined in Fig. 1.

### Sample size

We hypothesize that LITT will improve the overall survival without substantially compromising the health-related quality-of-life. The two co-primary outcomes were used to calculate the required sample size.

#### Overall survival

Recent analysis of the Dutch Quality Registry Neurosurgery data showed a median survival of patients with primary irresectable glioblastoma in the Netherlands of 5.1 months [9]. Current available literature states that LITT in newly diagnosed, irresectable glioblastoma may improve the median survival to 10.2 months [12]. A doubling of current survival to 10.2 months (median survival benefit of 5.1 months) is considered relevant, based on input from experts, the Dutch Society for Neuro-Oncology, and the Dutch patient organization.

A Cox proportional hazards model (two-sided; alpha = 0.05; power = 90%) was used for sample size calculations. A hazard ratio of 0.5 was assumed for overall survival (equivalent to an improvement in median overall survival from 5.1 to 10.2 months). The null hypothesis assumed a hazard ratio of 1. The allocation ratio will be 1:1. With 46 months of accrual and 18 months of follow-up, after an average follow-up of 41 months we will have reached 96% of events (overall probability of event, pE = 0.96). The calculated sample size therefore is 91. To account for a 5% post-randomization exclusion rate due to non-glioma diagnosis on histopathology, a 5% drop-out ratio prior to intervention and 10% loss to follow-up, 114 patients (57 per arm) need to be included.

#### Quality-of-life

HR-QoL will be evaluated as non-inferiority of EORTC QLQ-C30 + BN20 score after 5 months in those patients still alive at that time. This time point will reflect the estimated median survival in the control group, leaving at least 50% of patients to be analysed. In addition, at this timepoint a short-term treatment effect is expected to be mitigated, while a lasting effect could still affect QoL. Non-inferiority of HR-QoL is considered at a maximum decline of 10 points (5% instrument scale). This decline is in range with the reported MCID, which is generally accepted as the limit between “minimal change” and “moderate change” [20–22]. Also, the Dutch Society for Neuro-Oncology and the patient association confirm this as a relevant and clinically acceptable lower limit. Based on literature a standard deviation of 22 is assumed [20].

Since the expected effect (either positive due to prolonged survival or negative due to treatment-related morbidity) of LITT on HR-QoL is unclear, expected mean values are assumed the same between both groups. Assuming an estimated median survival time (MST) of 5.1 months in the control group vs. 10.2 months in the treatment group, the surviving proportion at 5 months after randomization (~ 4.5 months after intervention) is calculated by e^(-ln[2]/MST) *4.5 months. This yields a relative survival of 54.2% in the control arm and 73.6% in the treatment group. The sampling ratio (Ntreatment/Ncontrol) will then be 1.36 (0.73/0.54). To detect non-inferiority with 80% power at 0.05 one-sided significance level, we require a sample size of 52 in the control group at 5 months after randomization. Given the relative survival, we require 96 patients to be randomised to each group prior to treatment. Considering a 5% post-randomization exclusion, 5% drop-out prior to intervention and 10% loss to follow-up, 238 patients in total (119 per arm) need to be included.

#### Final sample size

The total sample size will be 238 patients (119 per arm) as this will enable us to study both survival and quality of life.

### Loss to follow-up and exclusion

Patients who withdraw informed consent, who must be excluded after randomization or are lost to follow-up due to another reason will be included in the intention to treat analysis and will not be replaced.

### Data collection and management

All data will be collected in an electronic data capture system (CastorEDC). Data will be retrieved from electronic patient files and electronic questionnaires. Local investigators will be trained in data collection. Twice a year a monitoring visit will take place at each research location to check and guarantee the quality of the data.

### Statistical methods

#### Primary outcomes

Survival analysis will be performed on an intention-to-treat basis. Cox proportional hazard analysis (two-sided) will be performed at 0.05 significance level, adjusted for treatment center, to evaluate the difference in survival between groups. Survival curves for each group will be estimated with the Kaplan-Meier method. Median survival differences with 95% confidence intervals (CIs) will be derived from the Kaplan-Meier estimates.

In patients alive at 5 months post-randomization, differences in EORTC QLQ-C30 + BN20 scores between the two treatment arms will be calculated using linear regression, adjusted for treatment center, and will be reported as mean difference with one-sided 95%CI. Non-inferiority p-values will be calculated.

#### Secondary outcomes

Cox proportional hazard analysis (two-sided) will be performed to evaluate the difference in progression-free and disease-free survival between groups. Kaplan-Meier estimates will be used to assess progression-free and disease-free survival.

Additional Cox proportional hazard analysis will be performed on overall, disease-specific, and progression-free survival measured from time of treatment, KPS and tumor size. Tumor volume response will be calculated as mean differences with 95%CIs. Complications will be calculated as rate difference with 95%CIs. Generic HR-QoL effects will be calculated as mean differences between groups with 95%CIs. Longitudinal effects will be assessed using mixed model analysis. Effects on adjuvant treatment will be calculated as proportions not completing CRT and mean differences in duration of CRT course with 95%CIs.

To assess the cost-effectiveness, an economic evaluation will be performed from both a healthcare and a societal perspective, adopting a 1.5-year time horizon. Costs will be calculated according to the Dutch guideline for economic evaluation, by multiplying resource use with the corresponding unit costs. Average costs will be calculated in both groups and differences will be calculated inclusive of 95% confidence intervals. Effectiveness will be measured in terms of quality-adjusted life years (QALYs). QALYs will be based on the utility scores as measured with the EQ-5D-5 L, using the Dutch tariff and the area under the curve method. If relevant, incremental cost-effectiveness ratios (ICERs) will be calculated by dividing estimated differences in costs by differences in QALYs. Uncertainty will be addressed by means of non-parametric bootstrapping and where relevant one-way sensitivity analyses are performed.

### Missing data

Missing data are common in end-of-life care studies since deterioration and/or death might lead to more cases of incomplete follow-up.

Therefore, multiple imputation procedures under missing at random (MAR) and missing not at random (MNAR) assumptions will be implemented and compared as a sensitivity analysis. If the results obtained under MAR and MNAR assumptions are similar, one can conclude that the presence of unobserved factors does not affect the conclusions [23].

#### Data Safety Monitoring Board (DSMB)

An independent DSMB is established to perform ongoing safety surveillance and to perform interim analyses on the safety data. None of the DSMB members has conflict of interest with the sponsor of the study.

#### (Serious) adverse events

All adverse events will be registered. Serious adverse events that result in death or that are life-threatening will be reported to the medical ethical committee (MEC) and DSMB within 7 days of notification. Other serious adverse events will be reported to the MEC and DSMB within 15 days. Adverse events which are possibly, probably, or definitely related to the surgical procedure will be reported to the DSMB within 15 days. All adverse events will be reported to the DSMB once every 6 months.

## Discussion

This trial will be the first multi-center randomised controlled trial assessing the (cost-) effectiveness of same-session LITT in primary irresectable glioblastoma. The trial will be performed in seven Dutch neurosurgical centers which in total cover the largest part of the Dutch neurosurgical patients. Other centers can refer patients to one of the participating centers when they consider them eligible for the trial. Since most Dutch neurosurgical centers will be involved in this trial, we expect the targeted number of inclusions to be achievable within the intended time. LITT will be performed in two of the seven participating centers. This will help to increase the possibility of scheduling LITT in the shortest possible time frame. We feel confident this trial can answer the question of (cost-)effectiveness of same-session LITT for this indication, but we realize we will face some challenges.

As with all (new) surgical techniques, a learning curve in performing LITT cannot be precluded, i.e., the neurosurgeons performing this procedure may improve over time. To date, little is known about the learning curve and important surgical outcomes of this relatively new technique. Learning curves will be considered when analysing the data.

Furthermore, we decided to include all WHO grade 4 gliomas in this trial as well as patients with a glioblastoma that can technically be resected, but who wish no craniotomy. We acknowledge that this might have an influence on the outcomes. Nevertheless, we expect this to be equal in both study groups due to randomization However, to monitor this, a subgroup analysis can be performed.

The questionnaires that will be used to measure the quality of life and costs are validated tools. However, the results will strongly depend on the completeness and quality of the questionnaires, which is known and inherent in Patient Reported Outcome Measures (PROMs) studies. We acknowledge that measuring quality of life through self-completed questionnaires in an open trial may cause information bias. However, given the nature of the condition and its impact on the QoL of patients, we do not expect a significant bias. We expect that, due to the course of this progressive disease and its significant impact on a person’s functioning, more missing (questionnaire) data will occur during the participation of a study subject.

The aim of this trial is to demonstrate whether same-session LITT is (cost-)effective in primary irresectable glioblastoma in the context of the program “Veelbelovende Zorg” of the Dutch Healthcare institute. This program aims to conditionally reimburse promising but expensive new treatments within a trial setting, to allow for the collection of high-quality evidence on its effectiveness and cost-effectiveness, which informs a definite reimbursement decision. When LITT proves to be effective, thus improving overall survival without compromising on quality of life, and appears to be cost-effective, the treatment will be included as a new standard treatment for this patient population and will be included in the Dutch basic healthcare reimbursement package.

### Electronic supplementary material

Below is the link to the electronic supplementary material.

Supplementary Material 1

## Data Availability

Not applicable.

## Declarations

### Protocol amendments

Important protocol modifications will be resubmitted to the MEC and will be communicated to all investigators and trial participants as well as the DSMB and the Dutch Healthcare Institute.

### Dissemination policy

The results of the study will be disclosed unreservedly. Both positive and negative results will be submitted for publication to peer-reviewed journal. The results of the study will be presented at (inter)national meetings and will be shared with the patient association and study participants.

### Trial status

This study is in the process of recruiting participants as of the 8th of April 2022.

## Abbreviations

CE: Conformité Européenne
CI: Confidence Interval
EDC: Electronic Data Capture system
CRT: Chemoradiation Therapy
DSMB: Data Safety Monitoring Board
EORTC: European Organization for Research and Treatment of Cancer
EQ5D-5L: EuroQol 5 Dimensions ? 5 Levels
GBM: Glioblastoma
HR: Health Related
ICER: Incremental Cost-Effectiveness Ratio
iMCQ: iMTA Medical Consumption Questionnaire
iMTA: Institute for Medical Technology Assessment
iPCQ: iMTA Productivity Cost Questionnaire
LITT: Laser Interstitial Thermal Therapy
MAR: Missing At Random
MEC: Medical Ethical Committee
MNAR: Missing Not At Random
MR: Magnetic Resonance
OS: Overall Survival
PIF: Patient Information Form
PROM: Patient Reported Outcome Measures
QALY: Quality-Adjusted Life Year
QLQ-BN20: Quality of Life Questionnaire Brain 20
QLQ-C30: Quality of Life Questionnaire Cancer 30
QoL: Quality of Life
RCT: Randomized Controlled Trial
SLA: Stereotactic Laser Ablation
USA: United States of America
WHO: World Health Organization
