# Supplementary material for: Study protocol for a multicenter randomised controlled trial on the (cost)effectiveness of biopsy combined with same-session MR-guided LITT versus biopsy alone in patients with primary irresectable glioblastoma (EMITT trial)
Source: BMC Cancer. 2023 Aug 23;23:788. doi: 10.1186/s12885-023-11282-7 (PMC10463911; doi:10.1186/s12885-023-11282-7)
Supplement: Supplementary file 1 — Supplementary Material 1 [file 12885_2023_11282_MOESM1_ESM.docx]

**APPENDIX**

**Supplementary item 1: list of participating centers**

Radboud University Medical Center (Radboudumc), Nijmegen

University Medical Center Utrecht (UMCU), Utrecht

Maastricht University Medical Center (MUMC+), Maastricht

Elisabeth-TweeSteden Ziekenhuis (ETZ), Tilburg

Erasmus Medical Center (Erasmus MC), Rotterdam

Amsterdam University Medical Center (Amsterdam UMC), Amsterdam

University Medical Center Groningen (UMCG), Groningen
